# Relationship Between Physical Exercise and Cognitive Function Among Older Adults in China: Cross-Sectional Population-Based Study

**DOI:** 10.2196/49790

**Published:** 2024-05-30

**Authors:** Fubaihui Wang, Changqing Gao, Yantao Wang, Zhuo Li, Feiran Zheng, Yanan Luo

**Affiliations:** 1 Social Science of Sport Research Center China Institute of Sport Science Beijing China; 2 Mental Health Center Kunming Medical University Kunming China; 3 Institute for Crime Prevention, Ministry of Justice Beijing China; 4 School of Social Sciences Tsinghua University Beijing China; 5 School of Ethnology and Sociology Minzu University of China Beijing China; 6 Department of Global Health School of Public Health Peking University Beijing China

**Keywords:** cognitive function, exercise, physical activity, mindfulness, cognitive exercise, mind stimulation, dementia treatment, cognitive intervention, cognitive treatment

## Abstract

**Background:**

The existing literature reveals several significant knowledge gaps that hinder health care providers in formulating exercise prescriptions for cognitive health.

**Objective:**

This study endeavors to elucidate the relationship between the level of physical activity and cognitive function in older adults in China. Moreover, it seeks to explore the associations between distinct exercise behaviors—such as exercise types, the purpose motivating engagement in exercise, the accessibility of exercise fields, and the inclination toward exercise—and cognitive function.

**Methods:**

Using data from the China Longitudinal Aging Social Survey (CLASS conducted in 2016, cognitive function was meticulously assessed through the modified Chinese version of the Mini-Mental State Examination, encompassing measures of orientation, memory, and calculation. Using self-report structured questionnaires, a myriad of information about physical activity during leisure time, exercise engagement, exercise intensity, primary exercise types, reasons for exercise participation, availability of sports facilities, and exercise willingness was diligently gathered. Robust ordinary least squares regression models were then used to compute coefficients along with 95% CIs.

**Results:**

A discernible inverted U-shaped trend in cognitive scores emerged as the level of physical activity surpassed the threshold of 500 metabolic equivalents of task (MET) minutes per week. Notably, individuals with a physical activity level between 500 and 999 MET minutes per week exhibited a coefficient of 0.31 (95% CI 0.09 to 0.54), those with a physical activity level between 1000 and 1499 MET minutes per week displayed a coefficient of 0.75 (95% CI 0.52 to 0.97), and those with a physical activity level above 1500 MET minutes per week demonstrated a coefficient of 0.45 (95% CI 0.23 to 0.68). Older individuals engaging in exercise at specific MET levels showcased superior cognitive function compared to their inactive counterparts. Furthermore, individuals driven by exercise motivations aimed at enhancing physical fitness and health, as well as those using sports facilities or public spaces for exercise, exhibited notably higher cognitive function scores.

**Conclusions:**

The findings underscore the potential of exercise as a targeted intervention for the prevention and treatment of dementia or cognitive decline associated with aging in older individuals. Leveraging these insights to formulate informed exercise recommendations holds promise in addressing a significant public health challenge linked to aging populations.

## Introduction

Cognitive function plays a crucial role within the framework of healthy aging proposed by the World Health Organization [1]. The decline in cognitive function can have adverse effects on social interaction, sense of purpose, independent living abilities, as well as the capacity to recover from illnesses or injuries [2,3]. Approximately 55.2 million people worldwide have cognitive impairments, and this number is projected to rise to 83.2 million by 2030 and further to 152.8 million by 2050 [4,5]. Given the rapid acceleration of global population aging, it has become imperative to identify and address potential cognitive decline. While cognitive decline is an inevitable part of the aging process, evidence suggests that cognitive plasticity can still occur, even in later stages of life [6]. The 2020 Lancet Commission on dementia prevention, intervention, and care indicates that approximately 40% of dementia cases worldwide are potentially modifiable [7], and a significant contribution can be attributed to engaging in physical activity and exercise can potentially reduce the risk of cognitive function [6,7].

Physical activity refers to any movement of the body that requires energy expenditure, such as gardening, shopping, or doing household chores [8], while exercise is a planned and structured form of physical activity that aims to improve or maintain physical fitness components [8]. Both physical activity and exercise have been extensively studied and shown to enhance cognitive performance through various molecular mechanisms, including the stimulation of brain-derived neurotrophic factors, learning, and memory [9,10]. As a result, clinical guidelines and public health recommendations often emphasize the importance of incorporating physical activity and exercise as fundamental strategies for preventing and managing dementia [7,11]. The World Health Organization suggests that older adults should aim for 150-300 minutes of moderate-intensity aerobic activity per week or 75-150 minutes of vigorous-intensity aerobic activity, along with regular muscle-strengthening exercises involving major muscle groups at least 3 times per week [12].

However, the existing literature reveals several significant knowledge gaps that hinder health care providers in formulating exercise prescriptions for cognitive health. First, although some evidence suggests the minimum effective, optimal doses, and maximum safe threshold of physical activity to enhance cognitive function in older adults [13], most of these studies have focused on high-income countries rather than limited-income countries. This discrepancy poses a challenge when attempting to develop exercise prescriptions for cognitive health in limited-income regions. Second, there is a limited number of studies that have distinguished the diverse effects on cognitive function between general physical activity and planned, structured exercise behaviors. Additionally, only a few studies have examined the specific influence of exercise itself on cognitive function while considering the controlling factors of physical activity metabolic equivalents. Third, there is insufficient evidence to determine the most effective types of exercise for improving cognitive function in older adults. Moreover, research is scarce regarding the relationship between different exercise behaviors, such as exercise purpose, chosen venue, exercise willingness, and cognitive function in older adults. However, this information concerning exercise behaviors plays a crucial role in guiding the development of exercise prescriptions aimed at enhancing cognitive health.

Therefore, to fill these gaps, this study used a nationally representative population-based study, the China Longitudinal Aging Social Survey (CLASS), to examine the relationship between exercise and cognitive function and its 3 domains among old adults. Here, we have included the assumptions of this study: first, we assume that there is a statistically significant relationship between the level of physical activity and cognitive function among older adults in China, and then, we assume that the aforementioned relationship will exhibit significant variations across different specific exercise behaviors (including various exercise types, the purpose for engaging in exercise, the availability of exercise fields, and the willingness of exercise).

## Methods

### Study Design and Participants

For this study, data were obtained from the CLASS conducted from 2014 to 2020. CLASS is a nationally representative survey that encompasses 28 provinces in China. It was designed by the Institute of Gerontology and implemented by the China Survey and Data Center at Renmin University of China. The survey used a stratified multistage probability sampling method to obtain participants from communities which involved 134 counties and 462 communities across the county in China. Participants aged 60 years and older were included in this study, and their demographic information, family and community details, health status, social functioning, physical activity, and retirement information were examined. Specially trained interviewers collected all the information through face-to-face interviews. More comprehensive information about this study’s design of CLASS can be found in a previous publication [14].

In 2016, the collection of metabolic equivalents of task (MET) values and detailed exercise information was limited to that specific year, while cognitive function and other covariates were collected across all 4 waves of data collection. As a result, our analytic sample includes individuals who responded to MET, detailed exercise information, cognitive function, and other covariates in 2016. In the 2016 survey, a total of 11,492 participants aged 60 years and older were involved. Among them, 2027 participants had missing data on cognitive function scores, 18 were missing information on the intensity of physical activities, 63 were missing information on exercise behaviors, and 186 were missing information on covariates. Individuals with missing values were excluded from our sample, resulting in a final analysis involving 9198 participants.

### Measures

#### Cognitive Function

The cognitive function of the respondents, including orientation, memory, and calculation, was assessed using the modified Chinese version of the Mini-Mental State Examination (MMSE). Orientation was evaluated using 5 questions that required respondents to provide information such as the current date, the village’s name, the date of National Day, the name of the current president, and the lunar calendar year. Each correct answer was assigned a score of 1, while incorrect answers received a score of 0, resulting in a range of 0 to 5 for orientation. Memory function was measured through immediate and delayed word recall tasks, where respondents were asked to recall 3 simple Chinese words immediately and several minutes later, respectively. The scores for immediate and delayed recall were combined, resulting in a range of 0 to 6. Calculation ability was assessed by having respondents count backwards from 100 by 7 seconds for 5 consecutive times, yielding a score ranging from 0 to 5. The sum of these 3 dimensions provided a representation of respondents’ cognitive functioning, with higher scores indicating better cognitive performance. According to a previous study [15], respondents who correctly answered at least three questions related to orientation were considered to have the cognitive ability to complete the full MMSE test. Therefore, the final cognitive functioning score ranged from 3 to 16.

#### Physical Activity

The self-report questionnaires were used to collect information on the extent of physical activity during leisure time. The questionnaire consisted of 9 questions that aimed to measure the time taken for different levels of activity, establishing based on the standard MET, which is a physiological measure expressing the energy cost of physical activities. It represents the ratio of the rate of energy expended during a specific physical activity to the rate of energy expenditure at rest. Further, 1 MET is defined as the energy expenditure at rest, typically around 3.5 mL of oxygen per kilogram of body weight per minute (3.5 mL O^2^/kg/minute). In practical terms, MET values are used to quantify the intensity of various activities. This study translated physical activities to METs involved assigning MET values to various activities based on the standard MET scale according to a previous study [16]. In our study, physical activities, such as light-intensity activities (including reading, playing an instrument, working on a computer, or walking at a slow or leisurely pace), moderate-intensity activities (including brisk walking, slow cycling, or playing tennis doubles), and vigorous-intensity activities (including jogging or running, bicycling, climbing briskly up a hill, or participating in an aerobics class), were involved. MET minutes per week were derived by quantifying the energy expenditure of different activities in terms of MET values and summing these values based on the duration of each activity. According to that, the level of leisure-time physical activity in this study was then categorized into several groups based on the calculated energy expenditure. The categories included: 0 (indicating a completely sedentary lifestyle), less than 500 MET minutes per week, 500 to 999 MET minutes per week, 1000 to 1499 MET minutes per week, and greater than 1500 MET minutes per week.

#### Exercise Behaviors

We investigated the relationship between exercise behaviors and cognition by examining aspects including whether participants engaged in exercise, the intensity of the exercise, the primary types of exercise, the reasons for engaging in the exercise, the availability of a sports field, and the willingness to participate in the exercise. These aspects were measured by (1) exercise: the presence of exercise behavior was measured by asking the question, “have you participated in physical exercise in the past year?”; (2) the intensity of exercise: the intensity of exercise was assessed using the questions, “how many days per week do you engage in high-intensity physical exercise that lasts at least 10 min and significantly increases your heart rate and sweating?” (frequency of high-intensity exercise per week) and “how many days per week do you engage in moderate-intensity physical exercise that lasts at least 10 min, increases your heart rate, and causes slight sweating?” (frequency of moderate-intensity exercise per week); these variables were measured as continuous variables; (3) the primary types of exercise: the primary types of exercise were determined by asking the question, “what types of physical exercise do you regularly participate in?” and categorized as follows: without exercise, endurance and speed exercises (eg, walking, running, swimming, cycling, badminton, and table tennis), apparent aesthetic exercises (eg, aerobics, dance, martial arts, and yoga), and other; (4) reasons for engaging in exercise: the reasons for participating in exercise were assessed by asking, “why do you engage in physical exercise?” and categorized as follows: without exercise, physical fitness and health enhancement (eg, disease prevention, maintaining physical fitness, and improving health), no specific purpose (ie, engaging in exercise without a specific goal and just for the sake of being active), and other (eg, self-realization and encouragement from children to be active); (5) the availability of sports fields: the availability of sports fields was measured by asking, “have you used the following sports facilities?” and categorized as follows: without exercise, athletic fields (eg, public sports fields and company sports facilities), parks, squares, or community fitness equipment (eg, parks, squares, residential area open spaces, and community fitness equipment), and home’s yard or curbside (eg, roadside and personal residence); (6) the willingness to participate in exercise: the willingness to engage in exercise was measured by asking, “if suitable conditions were provided for your exercise, would you be prepared to participate in exercise?” and categorized as “without willingness,” “uncertainty,” and “willingness.”

#### Covariates

Age (continuous covariates), gender (men or women), marital status (living without a spouse or living with a spouse), residency (urban or rural), education level (illiteracy, primary school, junior high school, senior high school, and above), working for pay (no or yes), household wealth (Renminbi, yuan), activities of daily living (ADL) impairment (no or yes), and having cardiometabolic disease (no or yes) were involved as covariates. Of these, cardiometabolic disease was consistent with the combination of diabetes, heart disease, and stroke according to previous studies [17-21], which was determined through the self-reported history obtained through an in-person visit with study personnel via a questionnaire.

### Statistical Analysis

Descriptive statistics are presented as means (SDs) for numerical variables and as numbers (%) for categorical variables. Ordinary least squares regression analysis was used to examine whether there is a relationship between the intensity of physical activity and cognitive function which was to calculate β coefficient and 95% CIs while adjusting for various factors including age, sex, marital status, residency, education level, occupation, household wealth, ADL impairment, and presence of cardiometabolic disease. Additionally, we use 8 ordinary least squares regression models to verify the associations between specific exercise behaviors and cognitive function. To examine the robustness of our findings, we conducted a subgroup analysis to explore the association between exercise and cognitive function among different subgroups. Specifically, we analyzed the male and female subgroups, individuals residing in rural and urban areas, individuals with low or high education levels, and individuals with or without cardiometabolic disease. All statistical analyses were performed using Stata (version 15; Stata Corp).

### Ethical Considerations

The data collection in CLASS was issued by the Biomedical Ethics Review Committee of Peking University (IRB00001052-11015). All participants were compensated and ethical approval for collecting data on human participants was received and updated annually at Peking University Institutional Review Board.

### Equity, Diversity, and Inclusion Statement

Our study embraces diversity by examining the relationship between exercise and cognitive function specifically among Chinese older adults. By focusing on this population, we contribute to the broader understanding of the benefits of exercise across diverse demographics. We strive for equity by ensuring equal representation and access to our study, regardless of socioeconomic background or other factors. We acknowledge that equitable opportunities for participation are essential in producing meaningful and inclusive findings. Inclusion is at the heart of our research, as we value the experiences, perspectives, and contributions of all individuals involved. We recognize that diversity and inclusion enhance the validity and applicability of our findings, promoting a more comprehensive understanding of the topic.

## Results

### Characteristics of Participants

Table 1 displays the characteristics of participants aged 60 years and older in China. The mean age of the participants was 69.55 (SD 7.21) years. Among the participants, 51.84% (n=4768) of them were men, 50.3% (n=4627) of them resided in urban areas, and 74.36% (n=6840) of them lived with their spouse. Regarding educational background, 23.05% (n=2120) of individuals were classified as illiterate and 13.71% (n=1261) of them were engaged in paid employment. The average household wealth was CN ¥241,267 (SD CN ¥425,622 [US $33,385.6, SD US $58,895.9]). Additionally, 5.97% (n=1414) of participants exhibited ADL impairment and 38.3% (n=3523) had cardiometabolic diseases.

The average global cognition score was 13.47 (SD 3.23), with specific mean scores of 4.67 (SD 0.78) for orientation, 4.90 (SD 1.67) for memory, and 3.90 (SD 1.72) for calculation. Among the participants, 16.37% (n=1507) of them reported engaging in exercise within the past year. On average, participants dedicated 0.20 (SD 0.94) days per week to high-intensity physical exercise lasting at least 10 minutes, which substantially increased heart rate and sweating. Additionally, participants allocated an average of 0.27 (SD 1.07) days per week to moderate-intensity physical exercise. Regarding the primary types of exercise, 14% (n=1288) of participants engaged in endurance and speed exercises, 1.89% (n=174) of them were involved in apparent aesthetic exercises, and 0.49% (n=45) of them fell into other categories. Furthermore, 12.95% (n=1191) of participants exercised for physical fitness and health enhancement, while 1.34% (n=123) of them had no specific purpose for engaging in exercise. In terms of available sports field facilities, 5.74% (528) of individuals had access to athletic fields when participating in exercise, 8.56% (n=787) of them could use parks, squares, or community fitness equipment, and 2.09% (n=192) of them used their home’s yard or curbside. As for willingness to engage in exercise, 40.41% (n=3717) of them expressed uncertainty, while 41.19% (n=3789) of them confirmed their readiness to participate in exercise if suitable conditions were provided.

**Table 1:** Characteristics of participants aged 60 years and above in China, 2016 (N=9198).

Variables			Values
**Cognitive function scores, mean (SD)**			
	Global cognition	13.47 (3.23)	
	Orientation	4.67 (0.78)	
	Memory	4.90 (1.67)	
	Calculation	3.90 (1.72)	
**Physical activity, n (%)**			
	Totally sedentary	954 (10.37)	
	<500 MET^a^, minutes per week	1603 (17.43)	
	500-999 MET, minutes per week	2275 (24.73)	
	1000-1499 MET, minutes per week	2024 (22.01)	
	≥1500 MET, minutes per week	2342 (25.47)	
**Exercise, n (%)**			
	No	7691 (83.63)	
	Yes	1507 (16.37)	
Frequency for high-intensity exercise, per week, mean (SD)			0.20 (0.94)
Frequency for middle-intensity exercise, per week, mean (SD)			0.27 (1.07)
**Main type of exercise, n (%)**			
	Without exercise	7691 (83.62)	
	Endurance and speed	1288 (14)	
	Apparent aesthetic	174 (1.89)	
	Other	45 (0.49)	
**Reasons for exercise, n (%)**			
	Without exercise	7691 (83.62)	
	Physical fitness and health enhancement	1191 (12.95)	
	No specific purpose	123 (1.34)	
	Other	193 (2.1)	
**Availability of sports field, n (%)**			
	Without exercise	7691 (83.62)	
	Athletic field	528 (5.74)	
	Park, square, or community fitness equipment	787 (8.56)	
	Home yard or curbside	192 (2.09)	
**Willingness to exercise, n (%)**			
	Without willingness	1692 (18.4)	
	Uncertainty	3717 (40.41)	
	Willingness	3789 (41.19)	
Age (years), n (%)			69.55 (7.21)
**Gender, n (%)**			
	Men	4768 (51.84)	
	Women	4430 (48.16)	
**Marital status, n (%)**			
	Living without spouse	2358 (25.64)	
	Living with spouse	6840 (74.36)	
**Residency, n (%)**			
	Urban	4627 (50.3)	
	Rural	4571 (49.7)	
**Education, n (%)**			
	Illiteracy	2120 (23.05)	
	Primary school	4083 (44.39)	
	Junior high school	1967 (21.39)	
	Senior high school and above	1028 (11.18)	
**Working for pay, n (%)**			
	No	7937 (86.29)	
	Yes	1261 (13.71)	
Household wealth (RMB^b^, CN ¥)^c^			241,267 (425,622)
**ADL^d^ impairment, n (%)**			
	No	7784 (94.03)	
	Yes	1414 (5.97)	
**Having cardiometabolic disease, n (%)**			
	No	5675 (61.7)	
	Yes	3523 (38.3)	

^a^MET: metabolic equivalents of task.

^b^RMB: Renminbi.

^c^US $33,385.6 (US $58,895.9).

^d^ADL: activities of daily living.

### Distribution of Cognitive Function Scores According to Physical Activity Levels and Exercise Behaviors

**Figure 1:**
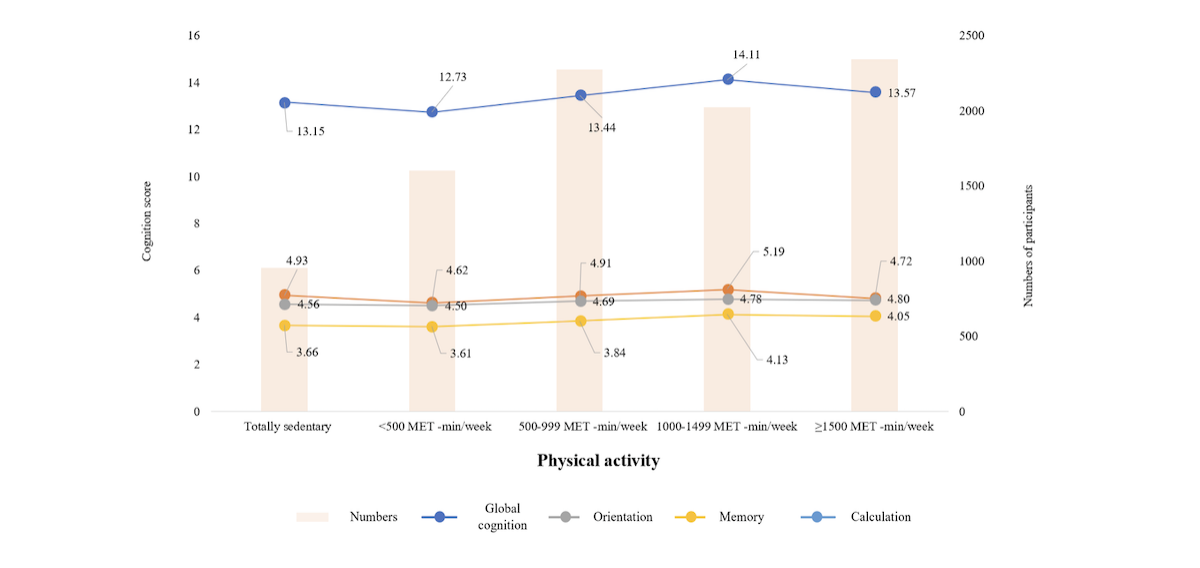
Distribution of cognition score according to physical activity levels.

Figure 1 shows the distribution of cognitive function scores according to physical activity levels. While participants with physical activity levels below 500 MET minutes per week exhibited lower cognitive function scores compared to those who were completely sedentary, cognitive function scores increased as the physical activity level increased from 500 to 1499 MET minutes per week (13.44-14.11). However, cognitive function scores started to decline after reaching a physical activity level of 1500 MET minutes per week or higher (13.57). Similar patterns were found in scores of orientation, memory, and calculation.

**Figure 2:**
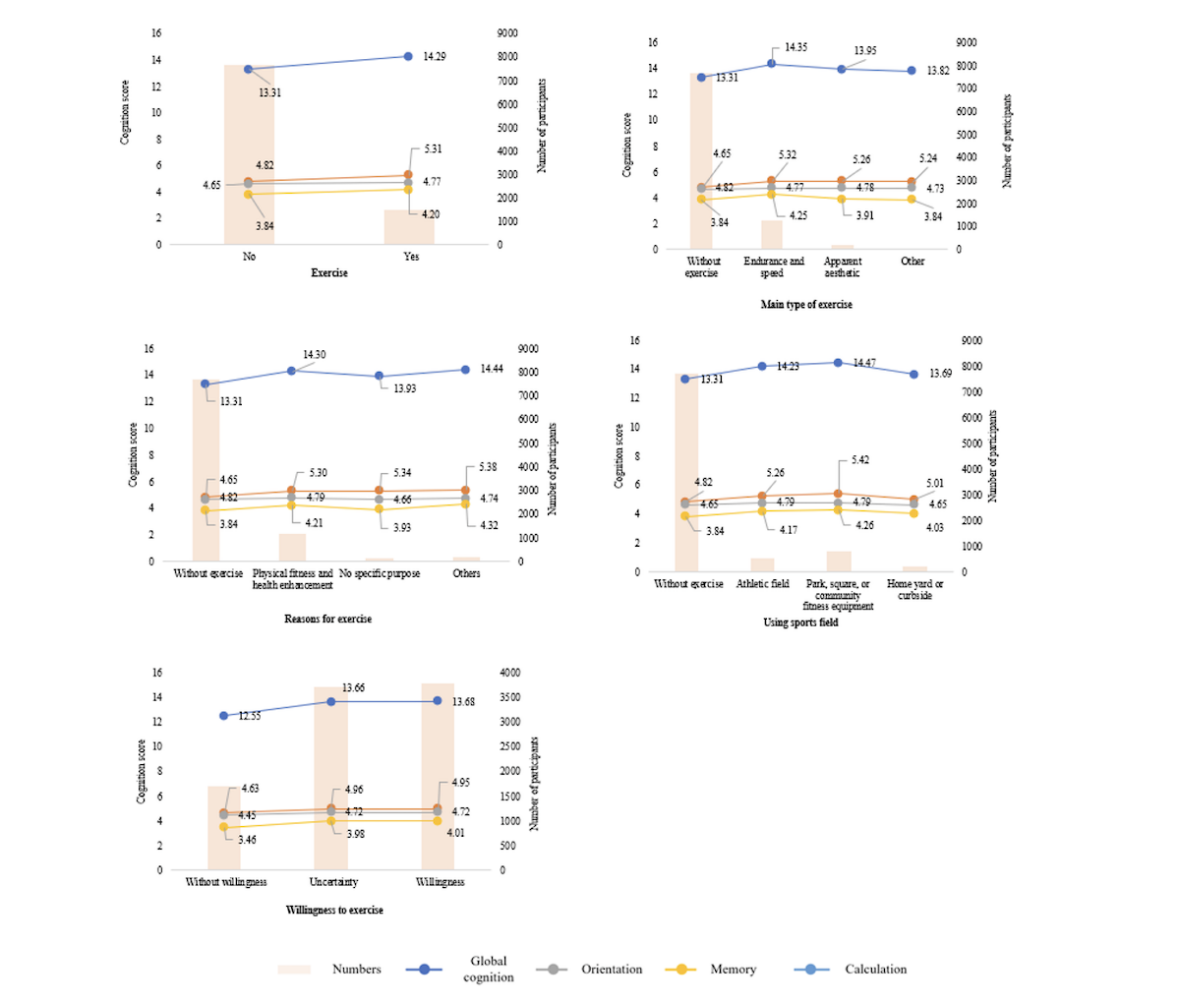
Distribution of cognition score according to exercise.

Regarding the scores of cognition based on the purpose of exercise, individuals with the purpose of exercise focused on physical fitness and health enhancement attained the highest cognitive function score (14.30). Conversely, individuals who exercised without a specific purpose had the lowest cognitive function score among those who engaged in exercise. Moreover, individuals who had access to athletic fields or parks, squares, or community fitness equipment exhibited higher cognition scores (>14.0) compared to those who exercised in their home’s yard or curbside. Lastly, individuals who expressed willingness to exercise if suitable conditions were provided had the highest cognitive function score (13.68), while those with uncertainty about exercise demonstrated a slightly lower score (13.66), and those without a willingness to exercise had the lowest cognitive function score (12.55). Comparable trends were observed in the cognitive domains of orientation, memory, and calculation. More specific details can be found in Figure 2 and Table S1 in Multimedia Appendix 1.

### Association Between Exercise Behaviors and Cognitive Function Among Older Adults in China

When compared to the cognition scores of individuals who were completely sedentary, there was no significant difference in the cognition scores of individuals with a physical activity level below 500 MET minutes per week (β=–0.04, 95% CI –0.28 to 0.20). However, as the physical activity level increased beyond the 500 MET minutes per week threshold, cognition scores showed an inverted U-shaped trend. The coefficient for individuals with a physical activity level between 500 and 999 MET minutes per week was 0.31 (95% CI 0.09 to 0.54), for individuals with a physical activity level between 1000 and 1499 MET minutes per week, the coefficient was 0.75 (95% CI 0.52 to 0.97), and for individuals with a physical activity level above 1500 MET minutes per week, the coefficient was 0.45 (95% CI 0.23 to 0.68).

Individuals who engaged in exercise within the past year exhibited higher levels of cognitive function compared to those without exercise engagement (β=0.49, 95% CI 0.32 to 0.66). Higher frequency of high-intensity or moderate-intensity exercise per week was associated with higher cognitive function scores (high-intensity: β=0.11, 95% CI 0.04 to 0.17; moderate-intensity: β=0.13, 95% CI 0.07 to 0.19). Specifically, individuals primarily involved in endurance and speed exercises had increased cognitive function compared to those without exercise (β=0.56, 95% CI 0.38 to 0.74). Participants who exercised to improve physical fitness or health demonstrated a coefficient of 0.49 (95% CI 0.30 to 0.68), whereas those with no specific purpose for exercise did not show a significant difference. Additionally, compared to individuals without exercise, the availability of athletic fields, parks, squares, or community sports facilities was associated with higher cognitive function scores. Individuals with exercise willingness or uncertainty if suitable conditions were provided exhibited higher cognitive function scores compared to those without exercise willingness (uncertainty: β=0.68, 95% CI 0.51 to 0.89; willingness: β=0.60, 95% CI 0.43 to 0.78). More details can be found in Table 2.

**Table 2:** Risk of cognitive function decline associated with exercise behaviors in China, 2016. The models were adjusted for age, sex, marital status, residency, education level, occupation, household wealth, ADL^a^ impairment, and having cardiometabolic disease or not.

Variables			Model 1		Model 2		Model 3		Model 4		Model 5		Model 6		Model 7		Model 8
**Physical activity, β coefficient (95% CI)**																	
	Totally sedentary	Reference		Reference		Reference		Reference		Reference		Reference		Reference		Reference	
	<500 MET^b^, minutes per week	–0.04 (–0.28 to 0.20)		0.92 (–0.05 to 1.89)		0.97 (0.00 to 1.94)		0.96 (–0.01 to 1.93)		0.92 (–0.05 to 1.89)		0.92 (–0.05 to 1.89)		0.94 (–0.04 to 1.91)		1.13 (0.16 to 2.10)	
	500-999 MET, minutes per week	0.31 (0.09 to 0.54)		1.00 (–0.89 to 2.88)		1.14 (–0.75 to 3.02)		1.12 (–0.76 to 3.01)		1.02 (–0.86 to 2.91)		1.00 (–0.89 to 2.88)		1.02 (–0.87 to 2.90)		1.31 (–0.58 to 3.19)	
	1000-1499 MET, minutes per week	0.75 (0.52 to 0.97)		1.79 (0.36 to 3.21)		1.81 (0.38 to 3.24)		1.80 (0.37 to 3.23)		1.81 (0.38 to 3.24)		1.79 (0.36 to 3.21)		1.82 (0.39 to 3.25)		2.04 (0.61 to 3.46)	
	≥1500 MET, minutes per week	0.45 (0.23 to 0.68)		0.66 (0.04 to 1.28)		0.72 (0.10 to 1.34)		0.70 (0.08 to 1.32)		0.66 (0.04 to 1.28)		0.66 (0.03 to 1.28)		0.65 (0.03 to 1.27)		0.85 (0.23 to 1.47)	
**Exercise, β coefficient (95% CI)**																	
	No	N/A^c^		Reference		N/A		N/A		N/A		N/A		N/A		N/A	
	Yes	N/A		0.49 (0.32 to 0.66)		N/A		N/A		N/A		N/A		N/A		N/A	
Frequency of high-intensity exercise, per week			N/A		N/A		0.11 (0.04 to 0.17)		N/A		N/A		N/A		N/A		N/A
Frequency of middle-intensity exercise, per week			N/A		N/A		N/A		0.13 (0.07 to 0.19)		N/A		N/A		N/A		N/A
**Main type of exercise, β coefficient (95% CI)**																	
	Without exercise	N/A		N/A		N/A		N/A		Reference		N/A		N/A		N/A	
	Endurance and speed	N/A		N/A		N/A		N/A		0.56 (0.38 to 0.74)		N/A		N/A		N/A	
	Apparent aesthetic	N/A		N/A		N/A		N/A		0.13 (–0.33 to 0.58)		N/A		N/A		N/A	
	Other	N/A		N/A		N/A		N/A		0.31 (–0.58 to 1.19)		N/A		N/A		N/A	
**Reasons for exercise, β coefficient (95% CI)**																	
	Without exercise	N/A		N/A		N/A		N/A		N/A		Reference		N/A		N/A	
	Physical fitness or health enhancement	N/A		N/A		N/A		N/A		N/A		0.49 (0.30 to 0.68)		N/A		N/A	
	No specific purpose	N/A		N/A		N/A		N/A		N/A		0.40 (–0.14 to 0.93)		N/A		N/A	
	Other	N/A		N/A		N/A		N/A		N/A		0.55 (0.12 to 0.99)		N/A		N/A	
**Availability of sports field, β coefficient (95% CI)**																	
	Without exercise	N/A		N/A		N/A		N/A		N/A		N/A		Reference		N/A	
	Athletic field	N/A		N/A		N/A		N/A		N/A		N/A		0.30 (0.03 to 0.57)		N/A	
	Park, square, or community	N/A		N/A		N/A		N/A		N/A		N/A		0.70 (0.48 to 0.93)		N/A	
	Home yard or curbside	N/A		N/A		N/A		N/A		N/A		N/A		0.23 (–0.20 to 0.66)		N/A	
**Willingness to exercise, β coefficient (95% CI)**																	
	Without willingness	N/A		N/A		N/A		N/A		N/A		N/A		N/A		Reference	
	Uncertainty	N/A		N/A		N/A		N/A		N/A		N/A		N/A		0.68 (0.51 to 0.86)	
	Willingness	N/A		N/A		N/A		N/A		N/A		N/A		N/A		0.60 (0.43 to 0.78)	

^a^ADL: activities of daily living.

^b^MET: metabolic equivalent of task.

^c^N/A: not applicable.

## Discussions

### Principal Findings

This study used nationally representative survey data to investigate the relationship between physical activity, exercise behaviors, and cognitive function among older adults in China. Our findings revealed that cognitive function was higher when the level of physical activity ranged from 500 to 1499 MET minutes per week. However, once the level of physical activity exceeded 1500 MET minutes per week, the degree of improvement in cognitive function diminished. These results present an opportunity for the development of future guidelines targeting physical activity in older adults to enhance cognitive abilities and may provide an achievable target (physical activity level ≥ 500 MET minutes per week) with substantial health benefits for many older adults. A meta-analysis suggested that exercise dose from 724 to 1200 MET minutes per week is associated with clinically relevant cognitive changes but with diminishing benefits observed beyond 1200 MET minutes per week [13]. The differences between our study and previous studies may be attributed to variations in the populations under investigation.

Our research has revealed that older individuals who participate in exercise exhibit higher cognitive function levels than those who are inactive, considering a specific level of MET for physical activity. This suggests that purposeful, organized, and planned exercise activities, in contrast to general physical activity, positively influence cognitive function. Physical exercise has been scientifically proven to decrease the risk of various negative health outcomes and positively impact individuals with mild cognitive impairment or dementia. These effects may be attributed to the elevation of neurotrophic proteins, reduction in the accumulation of harmful proteins, mediation of neuroinflammation, or inhibition of neuronal functional deficits [22-24]. Additionally, our findings indicate that endurance and speed exercises exert a more pronounced influence on cognitive function. This aligns with previous research that highlights the significant role of aerobic exercise and low-intensity resistance training in inducing clinically significant cognitive changes [13,25]. Our robust analysis was indicated by similar findings of the main results.

The results of our study reveal a robust link between exercise aimed at improving physical fitness and health and higher cognitive function scores. Furthermore, we noted that individuals who possess exercise motivation tend to exhibit higher cognitive function scores compared to those lacking motivation. These findings underscore the importance of emphasizing the impact of exercise on health through health education, boosting exercise motivation, and amplifying the cognitive benefits of exercise. Moreover, our research indicates a positive correlation between engaging in exercise in sports facilities or public spaces and achieving higher cognitive function scores. This suggests the potential advantages of constructing sports facilities or park areas in the future to maximize the benefits of exercise for the older adult population. In summary, our study represents a significant step toward providing precise exercise recommendations for enhancing the overall cognitive abilities of older individuals in China. It can serve as a valuable resource for developing tailored exercise plans and implementing patient-centered care approaches to enhance cognitive function in limited-income countries moving forward [26,27].

Our study is the first study to examine the specific associations of exercise itself (including the purpose of exercise, the choice of exercise place, and the willingness to exercise) with cognitive function while taking into account controls for the metabolic equivalent of physical activity. This study has several limitations. First, those with impaired cognitive function may have a limited capacity to exercise, which may lead to a reverse causal relationship between exercise and cognitive function. However, due to the cross-sectional design of this study, it could not exclude this influence. Subsequent research could use longitudinal designs to explore the bidirectional relationship and the causal impact of exercise behavior on cognitive function. Second, this study only incorporated respondents who completed cognitive function measurements in the CLASS survey, thereby excluding nonrespondents. This may result in an underestimation of the strength of the relationship between exercise and cognitive function. Future research should replicate this issue using a wider array of data sources. Third, due to data limitations, some important variables that could potentially impact cognitive function (such as depression, dementia, or Alzheimer disease) were not included in this study. Therefore, when interpreting our results, it is important to be mindful of this, and we hope that future research can use other data sources to further validate and delve deeper into this study’s findings. Fourth, the physical activity was measured through self-reporting based on the standard MET. Future studies should use more accurate measurement methods, such as accelerometer-measured relative physical activity intensity, to further validate our results. Fifth, this study used the MMSE to assess cognitive function, which may result in a ceiling effect for older adults. Future research should consider using other cognitive function measurement scales, such as the Montreal Cognitive Assessment, to validate our findings.

### Conclusions

Our results found that there was a statistically significant relationship between the intensity of physical activity and cognitive function among older adults in China. Our findings suggested that the optimal exercise dose associated with cognitive changes falls within the range of 500-1499 MET minutes per week. However, the benefits of exercise become less clear beyond 1500 MET minutes per week. Older individuals who engage in exercise at a specific level of MET for physical activity demonstrate higher levels of cognitive function compared to inactive individuals. Moreover, this relationship exhibited significant variations across different specific exercise behaviors: individuals with exercise motivation, aiming to improve physical fitness and health, and those who engage in exercise in sports facilities or public spaces, exhibit higher cognitive function scores than their counterparts. Our findings may offer practical guidance for health care professionals, enabling them to enhance cognitive health outcomes in older individuals through targeted exercise interventions and recommendations. Specifically, health care professionals should recognize that the impact of exercise motivation on cognitive function can be addressed through motivational strategies to encourage older individuals to engage in physical activity. Additionally, physicians or physiotherapists may need to advise older adults to monitor their exercise intensity within the specified range. This approach can optimize cognitive benefits while avoiding the potential diminishing returns associated with exceeding 1500 MET minutes per week. By using these findings to provide informed exercise recommendations, we can better address one of the significant public health challenges we face. Future studies can delve deeper into understanding the nuances of the optimal exercise dose associated with cognitive changes and use longitudinal designs to establish temporal relationships between exercise patterns and cognitive changes over time.

## Appendix

Multimedia Appendix 1 Supplementary Files.

## Abbreviations

ADL: activities of daily living
CLASS: China Longitudinal Aging Social Survey
MET: metabolic equivalent of task
MMSE: Mini-Mental State Examination

## Data Availability

The data sets generated or analyzed during this study are not publicly available owing to security protocols and privacy regulations but are available from the corresponding author upon reasonable request.
