# Supplementary material for: Relationship Between Physical Exercise and Cognitive Function Among Older Adults in China: Cross-Sectional Population-Based Study
Source: JMIR Public Health Surveill. 2024 May 30;10:e49790. doi: 10.2196/49790 (PMC11176873; doi:10.2196/49790)
Supplement: Multimedia Appendix 1 [file publichealth_v10i1e49790_app1.docx]

**Supplementary**

**Table S1.** Cognitive function scores by exercise behaviors among adults aged 60 years old and above in China

| **Variables** | **Global cognition** | **Orientation** | **Memory** | **Calculation** |
| --- | --- | --- | --- | --- |
| **Physical activity** |  |  |  |  |
| Totally sedentary | 13.15(3.29) | 4.93(1.68) | 4.56(0.99) | 3.66(1.88) |
| <500 MET -min/week | 12.73(3.72) | 4.62(1.88) | 4.50(0.99) | 3.61(1.86) |
| 500-999 MET -min/week | 13.44(3.25) | 4.91(1.72) | 4.69(0.72) | 3.84(1.73) |
| 1000-1499 MET -min/week | 14.11(2.74) | 5.19(1.39) | 4.78(0.58) | 4.13(1.55) |
| ≥1500 MET -min/week | 13.57(3.09) | 4.80(1.66) | 4.72(0.72) | 4.05(1.62) |
| **Exercise** |  |  |  |  |
| No | 13.31(3.30) | 4.82(1.72) | 4.65(0.81) | 3.84(1.76) |
| Yes | 14.29(2.68) | 5.31(1.33) | 4.77(0.61) | 4.20(1.46) |
| **Main type of exercise** |  |  |  |  |
| Without exercise | 13.31(3.30) | 4.82(1.72) | 4.65(0.81) | 3.84(1.76) |
| Endurance and speed | 14.35(2.65) | 5.32(1.33) | 4.77(0.60) | 4.25(1.42) |
| Apparent aesthetic | 13.95(2.74) | 5.26(1.38) | 4.78(0.68) | 3.91(1.65) |
| Other | 13.82(3.08) | 5.24(1.30) | 4.73(0.62) | 3.84(1.65) |
| **Reasons for exercise** |  |  |  |  |
| Without exercise | 13.31(3.30) | 4.82(1.72) | 4.65(0.81) | 3.84(1.76) |
| Physical fitness and health enhancement | 14.3(2.64) | 5.3(1.35) | 4.79(0.58) | 4.21(1.45) |
| No specific purpose | 13.93(3.33) | 5.34(1.34) | 4.66(0.88) | 3.93(1.71) |
| Other | 14.44(2.43) | 5.38(1.24) | 4.74(0.58) | 4.32(1.33) |
| **Availability of sports field** |  |  |  |  |
| Without exercise | 13.31(3.30) | 4.82(1.72) | 4.65(0.81) | 3.84(1.76) |
| Athletic field | 14.23(2.54) | 5.26(1.32) | 4.79(0.52) | 4.17(1.46) |
| Park, square or community fitness equipment | 14.47(2.62) | 5.42(1.22) | 4.79(0.60) | 4.26(1.42) |
| Home yard or curbside | 13.69(3.13) | 5.01(1.69) | 4.65(0.84) | 4.03(1.65) |
| **Willingness to exercise** |  |  |  |  |
| Without willingness | 12.55(3.75) | 4.63(1.84) | 4.45(1.05) | 3.46(1.95) |
| Uncertainty | 13.66(3.03) | 4.96(1.58) | 4.72(0.73) | 3.98(1.66) |
| Willingness | 13.68(3.10) | 4.95(1.68) | 4.72(0.67) | 4.01(1.63) |

**Table S2.** Subgroup analysis for the association between exercise behaviors and cognitive function among older adults aged 60 years old and above in China

| **Variables** | **Male** | **Female** | **Urban** | **Rural** | **Low education level** | **High education level** | **Without cardiometabolic disease** | **With cardiometabolic disease** |
| --- | --- | --- | --- | --- | --- | --- | --- | --- |
| **Exercise** |  |  |  |  |  |  |  |  |
| No | Reference | Reference | Reference | Reference | Reference | Reference | Reference | Reference |
| Yes | 0.51(0.28,0.74) | 0.40(0.16,0.65) | 0.38(0.19,0.56) | 0.65(0.34,0.96) | 0.39(0.18,0.61) | 0.58(0.34,0.83) | 0.48(0.27,0.70) | 0.41(0.14,0.68) |
| **Frequency for high intensity exercise, per week** | 0.09(0.01,0.18) | 0.10(0.00,0.20) | 0.13(0.05,0.20) | 0.06(-0.08,0.19) | 0.09(0.01,0.17) | 0.13(0.03,0.23) | 0.13(0.05,0.21) | 0.03(-0.08,0.14) |
| **Frequency for middle intensity exercise, per week** | 0.12(0.04,0.20) | 0.11(0.03,0.20) | 0.11(0.05,0.17) | 0.14(0.02,0.26) | 0.09(0.02,0.15) | 0.16(0.06,0.25) | 0.10(0.03,0.17) | 0.13(0.03,0.22) |
| **Main type of exercise** |  |  |  |  |  |  |  |  |
| Without exercise | Reference | Reference | Reference | Reference | Reference | Reference | Reference | Reference |
| Endurance and speed | 0.60(0.36,0.84) | 0.46(0.19,0.73) | 0.46(0.26,0.66) | 0.71(0.37,1.04) | 0.45(0.21,0.68) | 0.68(0.42,0.94) | 0.52(0.29,0.75) | 0.53(0.25,0.81) |
| Apparent aesthetic | -0.41(-1.22,0.41) | 0.19(-0.37,0.74) | 0.14(-0.36,0.63) | -0.09(-0.95,0.76) | 0.02(-0.51,0.55) | 0.09(-0.62,0.80) | 0.32(-0.23,0.87) | -0.50(-1.30,0.30) |
| Other | 0.27(-0.79,1.33) | 0.55(-1.01,2.10) | -0.76(-1.76,0.23) | 2.15(0.57,3.72) | 0.78(-0.32,1.88) | -0.06(-1.36,1.24) | 0.46(-0.64,1.55) | 0.28(-1.21,1.76) |
| **Reasons for exercise** |  |  |  |  |  |  |  |  |
| Without exercise | Reference | Reference | Reference | Reference | Reference | Reference | Reference | Reference |
| Physical fitness/health enhancement | 0.55(0.29,0.80) | 0.38(0.10,0.66) | 0.38(0.18,0.59) | 0.65(0.31,1.00) | 0.38(0.14,0.61) | 0.62(0.34,0.89) | 0.50(0.26,0.74) | 0.39(0.10,0.69) |
| No specific purpose | 0.22(-0.54,0.98) | 0.51(-0.24,1.26) | 0.48(-0.12,1.07) | 0.25(-0.73,1.23) | 0.38(-0.35,1.11) | 0.41(-0.32,1.14) | 0.57(-0.12,1.27) | 0.11(-0.73,0.95) |
| Others | 0.47(-0.14,1.07) | 0.47(-0.14,1.09) | 0.27(-0.18,0.72) | 0.99(0.08,1.90) | 0.52(-0.01,1.04) | 0.50(-0.15,1.15) | 0.30(-0.27,0.87) | 0.66(0.01,1.32) |
| **Availability of sports field** |  |  |  |  |  |  |  |  |
| Without exercise | Reference | Reference | Reference | Reference | Reference | Reference | Reference | Reference |
| Athletic field | 0.39(0.02,0.76) | 0.17(-0.21,0.56) | 0.14(-0.14,0.43) | 0.62(0.08,1.15) | 0.27(-0.06,0.60) | 0.32(-0.08,0.73) | 0.34(0.01,0.68) | 0.16(-0.28,0.61) |
| Park, square or community | 0.63(0.33,0.93) | 0.68(0.35,1.01) | 0.59(0.35,0.84) | 0.80(0.38,1.22) | 0.61(0.33,0.88) | 0.76(0.43,1.09) | 0.61(0.32,0.90) | 0.69(0.35,1.04) |
| Home yard or curbside | 0.41(-0.18,1.00) | 0.09(-0.53,0.71) | 0.20(-0.33,0.73) | 0.36(-0.32,1.04) | -0.16(-0.74,0.42) | 0.62(0.03,1.22) | 0.47(-0.07,1.01) | -0.09(-0.79,0.61) |
| **Willingness to exercise** |  |  |  |  |  |  |  |  |
| Without willingness | Reference | Reference | Reference | Reference | Reference | Reference | Reference | Reference |
| Uncertainty | 0.70(0.46,0.94) | 0.56(0.30,0.81) | 0.41(0.17,0.64) | 0.81(0.56,1.07) | 0.44(0.14,0.74) | 0.69(0.48,0.91) | 0.71(0.48,0.93) | 0.49(0.22,0.77) |
| Willingness | 0.55(0.31,0.79) | 0.60(0.34,0.85) | 0.44(0.20,0.67) | 0.65(0.40,0.91) | 0.24(-0.04,0.53) | 0.73(0.51,0.95) | 0.55(0.33,0.77) | 0.58(0.30,0.86) |

**Note:** all models adjusted for physical activity, age, sex, marital status, residency, education level, occupation, household wealth, ADL impairment and having cardiometabolic disease or not.
